# HSV Replication: Triggering and Repressing STING Functionality

**DOI:** 10.3390/v15010226

**Published:** 2023-01-13

**Authors:** Eric Krawczyk, Chase Kangas, Bin He

**Affiliations:** Department of Microbiology and Immunology, College of Medicine, University of Illinois, Chicago, IL 60612, USA

**Keywords:** herpes simplex virus, STING, interferon, viral replication, pathogenesis, antiviral immunity

## Abstract

Herpes simplex virus (HSV) has persisted within human populations due to its ability to establish both lytic and latent infection. Given this, human hosts have evolved numerous immune responses to protect against HSV infection. Critical in this defense against HSV, the host protein stimulator of interferon genes (STING) functions as a mediator of the antiviral response by inducing interferon (IFN) as well as IFN-stimulated genes. Emerging evidence suggests that during HSV infection, dsDNA derived from either the virus or the host itself ultimately activates STING signaling. While a complex regulatory circuit is in operation, HSV has evolved several mechanisms to neutralize the STING-mediated antiviral response. Within this review, we highlight recent progress involving HSV interactions with the STING pathway, with a focus on how STING influences HSV replication and pathogenesis.

## 1. Introduction

Herpes simplex viruses (HSV) are human pathogens responsible for a range of clinical manifestations. HSV-1 is the most common cause of infectious blindness and fatal encephalitis worldwide. It also accounts for an escalating number of newly acquired genital ulcers [1,2]. HSV-2 is commonly associated with genital herpes and encephalitis, which is a severe problem in neonates born to HSV-2-infected mothers [3]. During lytic infection, HSV enters the host cells and gains access into the nucleus where it releases its genome. The viral genome will then circularize and sequentially express sets of viral genes, categorized as immediate-early (IE), early (E), and late (L) genes that lead to the production of infectious virions. HSV typically targets epithelial cells of the mucosa to undergo lytic replication and subsequently penetrates to the peripheral neurons to establish latency. Viral reactivation occurs periodically, which is a lifelong source of infectious virus or recurrent lesions [4,5]. During this complex process, the virus triggers the antiviral response, resulting in the induction of proinflammatory cytokines, chemokines, and interferons (IFN) [6].

Mammalian cells encode multiple proteins that are capable of sensing HSV infection [7]. Pattern recognition receptors (PRR) recognize distinct danger signals in order to eliminate the pathogen [8]. These sensors detect pathogen-associated molecular patterns (PAMP), exemplified by aberrant DNA and RNA. Of note, several intracellular DNA sensors serve to activate the stimulator of interferon genes (STING), which mediates antiviral immunity [6]. Nevertheless, HSV encodes an array of antagonists, enabling viral replication or persistence. In this review, we will discuss recent progress on HSV infection pertinent to STING-mediated immunity.

## 2. Induction of STING Activity by HSV

HSV infection triggers STING [9,10], a highly conserved innate immune factor [11,12]. Within vertebrates, STING plays a critical role in the production of IFN. Additionally, STING facilitates the activation of autophagy and the transcription factor NF-kB [13]. STING contains 4 transmembrane helices that are connected to a cytoplasmic binding domain and a signaling domain [14]. As an ER-resident protein, STING is in a self-assembled dimer [15]. STING activation occurs upon binding to cyclic GMP-AMP (cGAMP), a second messenger synthesized by cGAMP synthase (cGAS), that senses intracellular double-stranded DNA in response to HSV infection [10]. In doing so, the STING dimer undergoes a confirmational change, with a 180 degrees rotation that exposes the C-terminal tail (CTT) to facilitate STING oligomerization [14,16,17,18]. This promotes its translocation from the ER to the Golgi apparatus (Golgi). This process involves multiple proteins [18,19]. The coat protein complex-II (COP-II) is responsible for creating a membrane vesicle that buds out of the ER, taking STING to the ERGIC [20,21,22]. In HSV-infected cells, the proteins TMED2 and iRhom2 and by association with TRAPβ, facilitates STING trafficking [20,23]. TMED2 specifically reinforces STING dimerization, whereas iRhom2 influences protein stability [20,23]. Within the ERGIC, the COP-II vesicles, together with STING, are sorted and transported out of the ERGIC. STING that stays with COP-II will go to the Golgi, whereas STING that transitions to COP-I will return to the ER [22]. 

**Figure 1:**
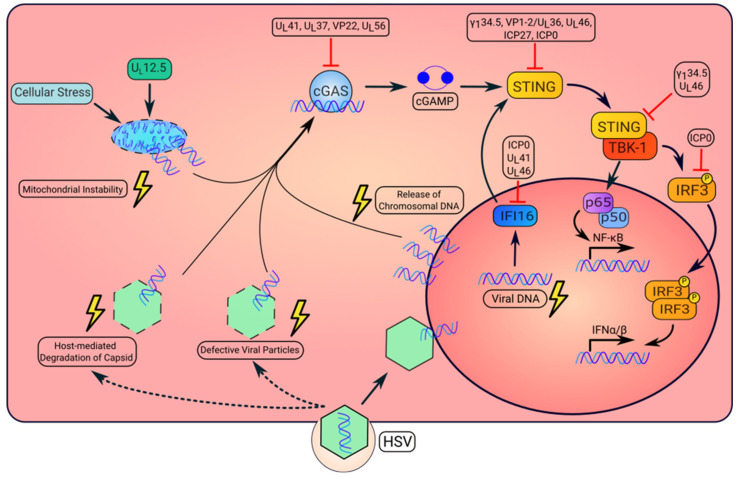
Activation and repression of the STING pathway by HSV. HSV infection can result in the altered localization of viral or dsDNA that is detected by intracellular DNA sensors. First, in the initial stage of infection, defective capsid can result in leakage of the viral genome into the cytosol. Additionally, proteasomal-dependent degradation of viral capsid may allow for release of the viral genome into the cytosol. Upon recognition of viral DNA, cGAS synthesizes the cyclic dinucleotide, cGAMP, that will bind to and activate STING. Once activated, STING recruits TANK-binding kinase 1, TBK1. This kinase complex phosphorylates the transcription factor, IRF3, resulting in its dimerization and nuclear translocation to induce type I IFN. STING also mediates the activation of NF-κB to produce inflammatory cytokines. Second, the virally encoded gene product UL12.5 can also induce mitochondrial instability resulting in release of mtDNA to the cytosol, which activates cGAS. Third, HSV replication causes the cytosolic release of host chromosomal DNA, which also activates STING signaling. Moreover, IFI16 senses viral DNA and moves to the cytoplasm where it activates STING. However, HSV expresses several proteins to inhibit STING pathway activation, which enables viral replication or pathogenesis (additional details on viral protein functions in Table 1).

At the Golgi, TRIM32 mediates K63-linked ubiquitination of the STING oligomer [24]. This is thought to enable STING to recruit TANK-binding kinase 1 (TBK1) via its PLPLRT/SD motif on the CTT [17,24], allowing for autophosphorylation of serine 172 within TBK1. Additionally, TBK1 phosphorylates the adjacent STING protein within the oligomer at the pLxIS motif found in the CTT [18,25]. Phosphorylation of both STING and TBK1 promotes a negative charge that recruits the interferon regulatory factor 3 (IRF3) molecule to bind to the pLxIS motif on STING [17,26]. Once phosphorylated by TBK1, IRF3 is released from the STING-TBK1 complex and translocated to the nucleus to activate IFN transcription (Figure 1) [27]. Consistently, phosphorylation of STING, TBK1, and IRF3 occurs in HSV infection leading to production of IFN [28,29,30]. Genetic deletion of STING, cGAS, or TBK1 compromises the antiviral response against HSV [10,29,31,32,33].

In addition to IRF3, STING activates the transcription factor nuclear factor-kappaB (NF-κB) during HSV infection [34,35]. Canonically, this is accomplished through phosphorylation of IкB by IKK complex which releases NF-кB transcription factors p50 and NF-кBp65/RelA [34]. After STING and TBK1 phosphorylation, TBK1 and IKKε can interact with TAK1 and the IKK complex [34,36]. NF-кB can also synergize with IRF3 in order to promote the transcription of IFN and proinflammatory cytokines [37]. HSV-1 infection stimulates the activation of NF-κBp65 along with reduced levels of IκBα [34,38,39]. There is also the involvement of TRAF6 upstream of TBK1 [34]. This interaction has been found to occur during DNA damage, where TRAF6 catalyzes the creation of K63-linked ubiquitin chains on STING [40].

Although incompletely understood, STING is also known to induce autophagy. Autophagy is an evolutionary conserved process that degrades invading pathogens as well as host proteins and organelles within the cell [41,42]. STING-induced autophagy is activated during STING translocation. STING-containing ERGIC vesicles are hypothesized to be able to deviate from the IFN production pathway to activate LC3 lipidation through a WIPI2 and ATG5-dependent mechanisms independent of ULK and the VPS34- kinase complex [41,43]. Following dsDNA stimulation, STING interacts with ATG9a and LC3 leading to autophagy [44]. Within HSV-infected cells, formation of LC3 punctum and conversion of LC3-I to LC3-II occurs [43]. This process is inhibited in STING knockout cell lines, supporting STING’s role in autophagy induction [41,43].

STING has been reported to activate inflammasome in HSV infection [45]. In order to accomplish this, STING orchestrates lysosomal cell death which in turn activates NOD-like receptor 3 (NLRP3) leading to caspase-1 activation and IL-1β release [46]. STING is thought to drive cytosolic-DNA-induced NLRP3 inflammasome activation through two mechanisms. First, the TM5 domain of STING interacts with NLRP3 through their NACHT or LRR domains, promoting NLRP3 localization to the ER and activating the inflammatory response. Second, STING deubiquitinates NLRP3 by reducing K48 and K63 polyubiquitination of NLRP3, thereby promoting inflammasome activation. These alternative functions of STING are vital to limit HSV infection although the underlying purpose awaits further investigation [45,46,47]. Collectively, these studies underscore the importance of STING in antiviral immunity in response to HSV infection.

### 2.1. Regulation of STING Activation by DNA Sensors

Several DNA sensors, including cGAS, the γ-interferon-inducible protein-16 (IFI16) and DDX41 activate STING in response to HSV infection [7]. Notably, cGAS localizes to both the cytoplasm and nucleus [48]. cGAS binds directly to either foreign or self-DNA, resulting in conformational changes in cGAS that allows for the optimal interaction between substrates ATP and GTP, allowing for synthesize 2′3-cGAMP [49]. The binding of DNA, additionally, leads to the formation of a liquid-like droplet which enhances the production of cGAMP [50].

IFI16, a PYHIN domain containing protein, recognizes HSV-1 viral DNA in the nucleus and upon activation localizes to the cytoplasm [51]. During early stages of infection, IFI16 interacts with the viral DNA directly through its HIN domain which in turn enhances cGAS-mediated cGAMP production and TBK1 recruitment to STING [52,53,54]. IFI16 is recruited to STING which leads to IFN production [53]. Knockdown of IFI16 in HFF decreases IFNβ gene expression [55]. Within the nucleus, IFI16 and cGAS interact with one another [52]. It is hypothesized that cGAS stabilizes IFI16 and increases cGAMP production. Whether this interaction promotes IFN production appears to be tissue-dependent [48,52,56,57].

DDX41 is a DExD/H-box helicase that binds to DNA and STING via its DEADc domain. This interaction triggers the recruitment of TBK1 and IFN production [58,59]. DDX41 also is reported to react to DNA virus infection from within the nucleus and translocate to the cytosol. Within the cytosol, DDX41 regulates cGAS by the annealing of ssDNA and unwinding of dsDNA [60]. Other DNA sensors, such as DNA-PK, have been found to interact with viral DNA and initiate an antiviral response [61,62,63,64,65]. RNA Polymerase III uses HSV dsDNA as a template to transcribe dsRNA which can then be recognized by RIG-I [66,67], which induces IFN through TBK1 and IRF3 [26,68]. While extensive work has been carried out to identify DNA sensors, a question that remains pertains to which type of DNA is the primary source that instigates STING pathway activation in the natural course of HSV infection.

### 2.2. Mechanisms of HSV Sensing

HSV infection depends on a number of glycoproteins to initiate viral entry. Upon attachment HSV fuses with either the plasma membrane or endosomal compartments to gain entry to the cytosol [4,5,69]. The viral nucleocapsid will then be transported to a nuclear pore, where the viral genome is injected into the nucleus [5]. Recent evidence has shown that mitochondrial, genomic, and viral DNA can trigger the antiviral response during HSV infection [8].

#### 2.2.1. Mitochondrial DNA

As metabolic hubs within the cell, mitochondria respond to numerous stimulants and are integrated into vital pathways such as programed cell death, redox homeostasis, and the antiviral response [70,71]. During HSV infection, mitochondrial protein production decreases dramatically [72,73]. Early work suggests that HSV disrupts the mitochondrial membrane and the release of enzymes into the cytosol [74]. Although it has been known that HSV affects mitochondrial function, current research supports the model that HSV can directly damage the mitochondrial DNA causing its release into the cytoplasm.

Cellular stress, brought on by viral infection, perturbs mitochondrial homeostasis leading to mitochondrial leakage and release of mitochondrial DNA (mtDNA) into the cytosol [75]. Additionally, HSV disrupts mitochondrial activity through the activity of a truncated form of the UL12 gene product, known as UL12.5 [71]. During HSV infection, UL12.5 localizes into the mitochondria causing a breakdown of mtDNA and alterations of the mitochondrial shape, resulting in release of mtDNA into the cytosol [71,74,76,77]. In response to viral infection, the mitochondria undergoes stress and leakage which releases mtDNA into the cytoplasm [77]. However, it is notable that unlike the cGAS activation of STING, RNA polymerase transcribes DNA into RNA that serves to activate the cytoplasmic RNA sensor RIG-I [76].

#### 2.2.2. Nuclear DNA

Within the nucleus, HSV DNA circularizes and subsequently leads to transcription and translation of viral products [5]. During viral replication, the nucleus architecture is reorganized to accommodate newly synthesized viral DNA and viral protein components required for the viral capsid [78,79]. This process results in massive mechanical stress to the nucleus causing disruption of the nuclear lamina and displacement of the host chromatin [78,79,80,81,82]. The loss of nuclear envelope integrity initiates the formation micronuclei, small DNA-containing envelope-like structures indicative of chromosome instability [83]. Prescence of micronuclei results in nuclear envelop collapse and cytoplasmic DNA sensor activation [56,84]. Mechanical stress induced by viral infection can trigger micronuclei formation and DNA fragments released into the cytoplasm due to genomic instability [56,85]. Nuclear DNA may also be released into the cytoplasm due to defects in DNA repair processing or nuclear envelope rupture, due to various stressors, which can cause activation of the innate immune response [56,85,86,87,88] HSV infection can lead to an accumulation of single and double stranded DNA breaks within the host DNA due to downregulations in DNA repair proteins such as Ku80 leading to neurodegenerative disorders [89]. However, when HSV-1 DNA is injected into the nucleus, there is elevated occurrences of chromatin stiffness and softening of the nuclear lamina to reduce nuclear DNA damage due to deformation [81,82]. These data support the view that HSV infection can result in nuclear DNA damage and that the nucleus lamina softens, and chromatin stiffens to minimize DNA damage. There is a possibility that DNA damaged by HSV infection could be released into the cytoplasm to activate the antiviral response.

#### 2.2.3. Viral DNA

HSV genomic DNA is contained within an icosahedral capsid that is surrounded by tegument proteins and a lipid envelope [6]. Following entry of the nucleocapsid, the icosahedral capsid is fully functional and able to transport the viral DNA to the nucleus. However, recent reports suggest the viral capsid is not always functional allowing DNA sensors to recognize HSV DNA [90,91]. During infection, a subset of HSV-1 DNA is released through an unknown mechanism. This event allows cGAS to gain access to the viral DNA. However, proteasome does not take part, although the process of IFN production is proteasome- and cGAS-STING-dependent [90]. Vp5 of the HSV capsid is also ubiquitinated resulting in proteasome degradation and exposure of viral DNA to the cytoplasmic dsDNA sensors [91]. Further exploration into this area is required to define whether this is a cell-type or tissue-dependent mechanism induced by HSV infection.

## 3. HSV Interference of the STING Pathway

STING is vital in the production of IFN, which exerts the antiviral response. However, HSV can persist within living organisms and establish latency. This is feasible because HSV gene products suppress STING pathway activation, summarized in Figure 1 and Table 1. Due to evolutionary pressures, HSV has evolved various viral functions that target the STING pathway, which facilitate productive viral replication and persistency.

### 3.1. ICP0

ICP0 is an IE gene that facilitates productive HSV replication and reactivation from latency. As a virus encoded E3 ligase, ICP0 activates the expression of the E and L HSV genes [92,93]. In HSV-infected cells, ICP0 confers viral resistance to IFN [94]. ICP0 interferes with DNA sensors, such as IFI16 and DNA-PK. HSV-1 mutants that lack ICP0 show an increase in replication and some viral gene expression in IFI16 depleted-cell lines compared with normal HFF cell line [95]. This is because ICP0 targets IFI16 through its RING finger domain [55]. This interaction facilitates the co-localization of ICP0 and IFI16 into nuclear punctate structures which allows for degradation of IFI16 to occur [96]. These observations indicate that silencing IFI16 impairs DNA sensing. Other studies suggest that ICP0 might regulate IFI16 in a cell-type-specific manner [96,97,98,99]. ICP0 can also interact with DNA-PK through its RING figure domain and cause degradation of the catalytic subunits DNA-PKcs [61,63].

Published work also suggests that ICP0 can modulate STING and IRF3. ICP0 can inhibit sustained activation of IRF3 within the cytoplasm or nucleus, through inhibition of IRF3 [55,100,101]. ICP0 has been reported to interact with STING, but whether the interaction benefits or harms HSV infection appears cell-type-dependent [102]. Cell types that have an impaired STING pathway, such as U2OS and Saos-2, are reported to promote the replication of HSV-1 with a deletion for the ICP0 gene [103]. The role these interactions play in HSV replication and establishment of infection is incompletely understood.

### 3.2. ICP27

ICP27 is an IE protein that is conserved within all herpesviruses [104]. ICP27 primarily regulates the E and L gene expression through a variety of mechanisms such as interacting with splicing factors to avoid mRNA splicing and the exporting of viral mRNA from the nucleus to the cytoplasm [104,105,106]. ICP27 is also reported to inhibit the type I IFN induction. Specifically, ICP27 interferes with the interactions between STING and TBK1 through the RGG motif in ICP27 [107]. This occurs downstream of TBK1 phosphorylation but upstream of IRF3 phosphorylation [107]. However, the impact of such protein–protein interactions on HSV replication has yet to be established.

### 3.3. UL36/VP1-2

VP1-2 is a tegument protein that is transcribed from UL36 gene N-terminal region [108]. VP1-2 promotes the release of the viral genome into the nucleus during infection [109]. The VP1-2 is a deubiquitinating enzyme found within HSV-1 [108]. This deubiquitinating activity is reported to prevent the host’s antiviral response [28,110]. In cell cultures, HSV-1 deficient in VP1-2 deubiquitinase activity displays decreased viral replication and increased IFN-β expression. This inhibition of IFN-β expression is reported to be due to VP1-2 deubiquitinating the K63-linked ubiquitin chains on STING preventing activation of IRF3 [28]. The data establish that VP1-2 ability to deubiquitinate is able to block host IFN production; however, the effect this process has on further antiviral processes requires further exploration.

### 3.4. UL37

UL37 is a tegument protein that is bound to the viral capsid. Along with UL36, UL37 is involved with intracellular transport of the viral capsid following viral entry and virion assembly and the development of the envelope capsid [111,112]. Deficient UL37 mutant HSV is impaired in its ability to replicate in vitro; however, deficiencies of cGAS within the L929 cell line and BMDMs permits replication recovery within this mutant virus. This evidence leads to the conclusion that UL37 disrupts IFN production [113]. UL37 is able to disrupt IFN production by impairing cGAS ability to catalyze cGAMP synthesis through deamidation of an asparagine residue found in human cGAS [113]. Although UL37 has been reported to interrupt cGAS functionality, it is possible that UL37 affects other PRR related to STING and IFN production.

### 3.5. UL41

UL41 encodes the virion host shutoff protein (vhs) that is an RNA endoribonuclease. It degrades mRNA which alleviates the cell’s antiviral response [114,115,116,117]. UL41 can promote the degradation of IFI16 in HeLa cells, independent of ICP0 through mRNA degradation [98]. Deficiency of UL41 in HSV triggers IFN production and decreases viral efficiency; however, the knockdown of cGAS in HFF cell lines rescues replication of UL41-deficient HSV. This is attributed to the inability of UL41-deficient HSV to degrade cGAS through RNases’ activity [114]. The primary function of UL41 is the elimination of host proteins through the degradation of host mRNA. Although this process is well established, the reason why UL41 degrades some host mRNA and leaves others is still debated [118].

### 3.6. UL46

UL46 is a tegument protein that accumulates late during viral infection [119]. During early infection, UL46 assists in the expression and regulation of transcriptional induction of IE genes, in association with VP16 [120]. In later stages of infection, UL46 facilitates virion assembly within the cytoplasm [121]. UL46-deficient viruses display deficient growth and upregulation of the innate immunity [28,122]. This is reversed in STING-deficient HEL and Hep-2 cell lines, suggesting that the UL46 protein negatively regulates the STING-dependent pathway [122]. UL46 can bind to both STING and TBK1 during HSV infection [122]. UL46 impact on TBK1 is related to UL46 ability to reduce dimerization of TBK1 leading to downregulation of IRF3 activation. Additional data suggest that UL46-deficient HSV-1 activates IRF3 and TBK1, whereas this is prevented during wild-type (WT) HSV-1 infection [119]. It was also reported that elimination of the IFI16 and STING proteins occurred in HEL, HEp-2, and HEK-293 cell lines that expressed UL46 protein [122]. These data together indicate that the interaction of UL46 with TBK1 and STING is a viable method to prevent IFN production.

### 3.7. VP22 (UL49)

VP22 is a tegument protein that is encoded by UL49 and is conserved within the subfamily *alphaherpesvirinae*. VP22 has many functions within infected cells such as reorganization of microtubules [123,124] and incorporation and transportation of RNA into uninfected cells [125]. VP22 is also known for disrupting IFN production through the cGAS-STING pathway. The VP22-deficient virus was unable to inhibit the activation of the IFN signaling pathway and reduced replication within HFF. Recovery of the VP22-deficient virus occurred when infecting in HFF cell lines that were cGAS knockdowns. This occurs due to the ability of VP22 to inhibit enzymatic activity within cGAS [126]. It has been demonstrated that VP22 can interfere with cGAS’s ability to bind to DNA through forming a liquid condensation with DNA disrupting cGAS activity [127]. VP22 inhibition of the antiviral response has recently been reported and much more research needs to be conducted to see if VP22 has any further effects on IFN production.

### 3.8. UL56

UL56 is a tegument protein whose primary role is the transportation and release of infectious virions, specifically within neurons [128,129]. UL56 recently has been reported to inhibit the antiviral response. HSV-1 deficient in UL56 triggers antiviral gene production. UL56-deficient HSV-1 replication is rescued in STING knockout cells. It is concluded that UL56 ability to inhibit cGAS from binding to DNA through direct interaction was the cause of these observations [130].

### 3.9. γ134.5

The γ_1_34.5 protein promotes viral replication in the peripheral tissues and neurovirulence [131,132,133]. While categorized as a leaky late gene product, γ_1_34.5 is detectable early in HSV infection [132,134,135]. The most well studied aspect of γ_1_34.5 is its ability to block protein synthesis shutoff conducted by PKR through eIF2α dephosphorylation [136,137,138]. However, γ_1_34.5 has additional functions, including regulation of ICP0 expression, interference in autophagy, dendritic maturation, and intracellular nucleic acid sensing [29,30,139,140,141,142]. Recent work suggests that γ_1_34.5 inhibits STING activation [30]. As such, unlike the wild-type virus, the HSV mutant devoid of functional γ_1_34.5 stimulates IFN production, which decreases viral replication. These Δγ_1_34.5 HSV viruses can recover replication when STING and TBK1 are deleted within the MEF cell line [29,30]. In HSV-infected HFF-1 cells, γ_1_34.5 interacts with STING, which prevents STING phosphorylation and translocation from the ER to Golgi apparatus. However, the precise mechanism by which this occurs is to be defined [30].

**Table 1:** Notable HSV antagonists of the STING pathway.

Viral Protein	Target Protein	Mechanism of Action	Reference
ICP0	IFI16	Interacts with and mediates IFI16 degradation	[55]
	DNA-PK	Targets DNA-PK for proteasomal degradation	[61]
ICP27	STING	Interacts with the STING-TBK1-IRF3 complex	[107]
VP1-2/UL36USP	STING	Removes the K63-like polyubiquitin from STING	[28]
UL37	cGAS	Deamidizes cGAS causing an impairment in cGAS ability to produce cGAMP	[113]
UL41	cGAS	Targets cGAS mRNA for degradation	[114]
	IFI16	Targets IFI16 mRNA for degradation	[55]
UL46	TBK1	Reduces dimerization of TBK1 impairing interaction with IRF3	[119]
	STING and IFI16	Presence of UL46 causes elimination of STING and IFI16	[122]
VP22	cGAS	Inhibits enzymatic activity of cGAS Forms a liquid condensation with DNA disrupting cGAS activity	[126] [127]
UL56	cGAS	Interacts with cGAS to inhibit binding to viral DNA	[130]
γ_1_34.5	STING	Interacts with and blocks STING translocation from ER to the Golgi	[30]

## 4. STING in HSV Replication and Pathogenesis

Accumulating evidence suggests that HSV interplay with the STING pathway dictates the outcome of infection in vivo. The STING pathway branches off into multiple antiviral responses, most of which have been reported to be inhibited by viral proteins HSV produces, resulting in productive infection.

HSV are able to replicate and establish latency within a variety of animal models [131,133]. However, STING functions to limit viral replication and dissemination. Accordingly, STING-knockout mice are more susceptible to lethal infection after infection with HSV-1 as compared to WT mice [32]. This is accompanied by decreased survival and type I IFN production upon intravenous or intracerebral HSV infection [143]. Similarly, STING-knockout mice exhibit profound neural invasion in ocular HSV infection [144,145]. It has been reported that STING in microglia orchestrates antiviral defense in the central nervous system [31]. Alternative mutations of STING that affect TBK1 binding, such as with L373A mutant or deletion of the CTT, in the mice are no longer able to mount an effective immune response when infected with HSV-1 [35]. However, the mice that have a point mutation of serine 365A, which only disrupts IFN production, within STING are still able to successfully resist HSV infection through an unknown mechanism independent of IFN production [47,146]. Yamashiro et al. proposed that this IFN-independent pathway occurs through STING-induced autophagy [47]. However, work by Yum et al. suggests that this may occur via NF-κB activation by STING [35]. What is determined is that STING is imperative to control HSV virus during HSV intravenous, ocular, or cranial infection [32,143].

Consistent with these observations, cGAS -/- mice are more susceptible to lethal infection [33]. It is reported that cGAS, alongside with STING, orchestrates the antiviral defense in the central nervous system. Defective cGAS in mice leads to increase susceptibility of acute encephalitis [31]. Within the vagina, mice succumbed to lethal HSV infection within cGAS-deficient mice [147]. However, DNA-PK has been reported to increase mice survival when inhibited [148]. This is assumed to be due to DNA-PK inability to regulate the immune system once it has been inhibited, similar to what has been found in patients with DNA-PK mutations [148,149]. Mice deficient in IRF3 had very little effect on HSV replication, whereas IRF7 and the IRF3 and IRF7 double-knockout cell lines displayed increased susceptibility to HSV corneal infection that were often fatal [150]. In humans, IRF3 and TBK1-deficient patients have been reported to have increased susceptibility to herpes-induced encephalitis (HSE) as well as an increase in reoccurrence of infection [151,152,153].

Several mutants of HSV have been reported to display different phenotypes in wild-type and STING-deficient mice. Although attenuated in wild-type mice, γ_1_34.5 null mutants are more virulent upon intracranial or intravenous infection of STING-deficient mice [143]. These mice displayed increased susceptibility to HSV infection and death. Another HSV mutant that lacks a functional UL36 exhibits elevated IFN induction and reduced growth in brain infection [28]. The UL37 mutant was able to replicate within infected mice that were STING- or cGAS-deficient with intraperitoneal injection [115]. However, other viral antagonists of the STING pathway proteins need to be pursued further in vivo.

## 5. Perspectives

HSV are large DNA viruses that interacts with the STING pathway in complex ways. Available evidence has established the importance of STING in protecting the host cells from HSV infection. Several intracellular DNA sensors coordinate with STING to detect HSV infection, where viral, mitochondrial, and chromosomal DNA can trigger the antiviral response. It is interesting to consider how these PRRs recognize HSV during natural infection, which might involve temporal or tissue specific events. Relevant to this is whether viral and host DNA contribute differentially in HSV life cycles. Work in recent years demonstrates that HSV has formulated numerous ways to successfully replicate within the host through exploitation of critical steps within the antiviral response. Remarkably, several HSV proteins negatively regulate the STING pathway. As these viral proteins belong to different kinetic class, a question arises as to whether they function cooperatively to provide an advantage to viral replication. The intricacies that occur during STING activation and how HSV effects the outcome needs to be further clarified. Further understanding of the interaction between STING and HSV may lead to better prophylactic or therapeutic interventions.
